# The Role of Cathepsin B in Peritoneal Fibrosis due to Peritoneal Dialysis

**DOI:** 10.1155/2019/4150656

**Published:** 2019-11-13

**Authors:** Su Ah Sung, Dong Hee Kim, Kook-Hwan Oh, Sang Youb Han, Kum Hyun Han

**Affiliations:** ^1^Department of Internal Medicine, Eulji Medical Center, Eulji University, Seoul 01830, Republic of Korea; ^2^Department of Surgery, Eulji Medical Center, Eulji University, Seoul 01830, Republic of Korea; ^3^Department of Internal Medicine, Seoul National University Hospital, Seoul 03080, Republic of Korea; ^4^Department of Internal Medicine, Inje University Ilsan Paik Hospital, Goyang-si, Gyeonggi-do 10380, Republic of Korea

## Abstract

Glucose-containing peritoneal dialysis (PD) solution causes peritoneal fibrosis (PF) characterized by accumulation of extracellular matrix (ECM) in the submesothelial layer. Cathepsin B is a lysosomal cysteine protease that degrades ECM, but its role in the PF remains unclear. Thus, we investigated the role of cathepsin B in PF. Procathepsin B was measured in the 73 PD effluents of 68 patients. Procathepsin B and cathepsin B after exposure of glucose and the effects of cathepsin B on the expression of matrix metalloproteinases (MMPs), tissue inhibitor of metalloproteinases (TIMPs), and urokinase-type plasminogen activator (uPA) were measured in the supernatant of cultured human peritoneal mesothelial cells (HPMCs). The effect of cathepsin B and its inhibitor, cystatin C, on PF was investigated in the murine model. Procathepsin B was measured at 3.6 *μ*g/L in serum and 5.4 *μ*g/L in PD effluent and positively correlated to the cancer antigen (CA) 125. The treatment with 4.25% glucose increased procathepsin B by 3.1-fold and cathepsin B by 5.9-fold in the HPMCs. Cathepsin B induced the secretion of MMP-1, -2, and -3 and TIMP-1 in the HPMCs, but uPA was not excreted. In the PF murine models, cathepsin B reduced the thickness of the submesothelial layer and cystatin C attenuated the effect of cathepsin B. HPMCs secrete cathepsin B with exposure of PD solution, and cathepsin B might help protect against PF.

## 1. Introduction

Peritoneal dialysis (PD) is one of the potential renal replacement therapies for ESRD, along with hemodialysis and kidney transplantation, and is the main treatment modality in some countries, such as Hong Kong, Colombia, and New Zealand [1]. The total number of ESRD patients receiving renal replacement therapy in 2015 was 87,014, including 7,352 patients in Korea receiving PD [2].

Ultrafiltration failure is the main reason for stopping PD and occurs in 36% of PD patients using nonphysiologic PD solutions over 5 years [3]. The morphopathologic characteristics of ultrafiltration failure include peritoneal fibrosis, with thickening of the submesothelial layer starting from as early as 2 years after initiation of PD to become 10 times thicker than the normal after 8 years in patients treated with nonphysiologic solutions [4]. Hypertonicity, acidic pH, high glucose concentrations, and glucose-degradation products have been suggested to be responsible for the development of peritoneal fibrosis [5, 6].

Peritoneal fibrosis occurs when extracellular matrix (ECM) synthesis exceeds its degradation. In terms of ECM production, many in vitro studies have revealed that human peritoneal mesothelial cells (HPMCs) excrete matrix proteins including collagens I, II, III, and IV, together with fibronectin, heparan sulfate, and dermatan sulfate proteoglycans, which are enhanced by high glucose stimulation [7–11]. In contrast, few studies have investigated ECM degradation in peritoneal fibrosis and most have concerned matrix metalloproteinases (MMPs) and their inhibitors, tissue inhibitors of metalloproteinases (TIMPs) [12–17]. There have been no studies of matrix proteases other than MMPs.

Cathepsin B is a potent lysosomal cysteine protease that has been extensively studied in the context of malignancies. Its expression is increased in various human cancer cells, and it plays a role in tumor invasion by degrading the ECM directly and via MMP/TIMP and urokinase-type plasminogen activator (uPA) pathways [18–22]. Cystatin C is used as a marker for renal function and is the most important extracellular inhibitor of cathepsin B [23]. However, the roles of cathepsin B and cystatin C in peritoneal fibrosis have not been investigated.

We aimed to investigate the role of cathepsin B in peritoneal fibrosis in PD patients. We measured procathepsin B and cystatin C levels in PD effluent and determined if cultured HPMCs secreted procathepsin B and cathepsin B in response to high glucose conditions. We also examined the effects of cathepsin B on the secretion of MMPs, TIMPs, and uPA in in vitro cultured HPMCs and on peritoneal fibrosis in a chlorhexidine-treated mouse model.

## 2. Materials and Methods

### 2.1. Patients, Clinical Parameters, and Measurement of Procathepsin B, Cystatin C, and Cancer Antigen (CA) 125

We enrolled consenting PD patients who underwent Kt/Vurea measurement and a peritoneal equilibrium test (PET) according to routine management protocols in two PD clinics of university-affiliated hospitals (Eulji Medical Center and Inje University Ilsan Paik Hospital) in Korea. Total Kt/Vurea and modified PET using 4.25% glucose dialysate were measured routinely in the PD clinics within 1 month after starting PD, 6 months later, and then every year. Patients with acute illnesses requiring admission or with acute bacterial infection, including peritonitis, within 1 month were excluded.

Twenty-four hours urine, unless the patient was anuric, and 24 hours PD effluent were collected from the patients. Renal and peritoneal Kt/Vurea was calculated using urea clearance data from a 24-hour collection of urine and PD effluent. The urea-distribution volume was calculated using the Watson equation [24]. Total Kt/Vurea was calculated as the sum of renal and peritoneal Kt/Vurea.

For PET, patients with an overnight dwell of 1.5% glucose dialysate were subjected to a 4-hour dwell with 4.25% glucose dialysate on the following morning. PD effluent samples were taken at 0- and 4-hour during the test, and blood samples were taken at 2-hour. The PD effluent/plasma ratio for creatinine (D/Pcr) was calculated and corrected for glucose interference using a correction factor derived by each center's laboratory. Cystatin C and procathepsin B were measured in the 2-hour blood samples during PET and the overnight PD effluent. Cystatin C was measured by nephelometry, and procathepsin B was measured by a quantitative sandwich enzyme immunoassay (R&D Systems, Minneapolis, MN, USA). We also measured CA 125 in the overnight PD effluent by electrochemiluminescence (Roche Diagnostics, Risch-Rotkreuz, canton of Zug, Switzerland). All protocols were approved by the Institutional Review Board of the Eulji Medical Center and Inje University Ilsan Paik Hospital (approval code: EMCIRB 08-37 and IB-0812-080).

### 2.2. HPMCs

Human omentum samples were obtained from consenting patients undergoing elective abdominal surgery, washed with sterile phosphate-buffered saline, and then incubated in 0.05% trypsin with 0.02% ethylenediaminetetraacetic acid solution for 20 minutes at 37°C. The suspension was centrifuged at 100 ×*g* for 10 minutes at 4°C, and the cell pellet was then washed and resuspended in Medium 199 (M199; Sigma-Aldrich, St. Louis, MO, USA) supplemented with 10% fetal bovine serum, L-glutamine 2 mM, penicillin 100 IU/ml, streptomycin 100 *μ*g/ml, hydrocortisone 0.4 *μ*g/ml, insulin 5 *μ*g/ml, and transferrin 5 *μ*g/ml. The cells were then seeded onto culture dishes and grown in the same medium at 37°C in humidified 5% CO_2_ in air. The culture medium was changed 24 hours after seeding and then every 3 days.

We investigated the effect of glucose on procathepsin B and cathepsin B secretion by HPMCs after incubating the cells in serum-free medium to arrest and synchronize cell growth. The cells were treated with M199 as a control (glucose 5.6 mmol/L), with M199 containing 1.5% glucose (addition of glucose 77.7 mmol/L), 4.25% glucose (addition of glucose 230.5 mmol/L), and their osmotic controls (addition of mannitol 77.7 mmol/L and 230.5 mmol/L, respectively) or lipopolysaccharide (LPS; Sigma-Aldrich) 5 *μ*g/ml. The concentration of procathepsin B and cathepsin B in the supernatant of HPMCs was measured by the sandwich enzyme immunoassay for procathepsin B and cathepsin B (AbFrontier, Seoul, Republic of Korea) 6 hours later.

We treated HPMCs with 1 and 5 ng/ml human cathepsin B (Sigma-Aldrich), respectively, and determined their effects on the secretion of MMPs, TIMPs, and uPA 12 hours later. The protein expression of MMP-1, MMP-2, MMP-3, TIMP-1, TIMP-2, and uPA in the supernatant was determined using enzyme-linked immunosorbent assay (ELISA) kits (MMPs, R&D Systems; TIMPs, EMD Millipore, Burlington, MA, USA; and uPA, R&D Systems). All protocols were approved by the Institutional Review Board of the Eulji Medical Center (approval code: EU 09-13).

### 2.3. Murine Peritoneal Fibrosis Model

C57BL/6 male mice (age 20 weeks and weight 20–30 g) were purchased from Orient Bio Company (Seongnam-si, Gyeonggi-do, Republic of Korea). Peritoneal fibrosis was induced using a solution of 0.1% chlorhexidine gluconate and 15% ethanol dissolved in saline. A total of 40 mice were divided into four groups (10 for each group). Ten mice were treated with daily intraperitoneal injections of 0.3 ml normal saline for 6 days (group 1). The other 30 mice were treated with daily intraperitoneal injections of 0.3 ml chlorhexidine solution for 3 days and were then divided into three groups and treated with normal saline 0.3 ml (group 2), 0.5 *μ*g/ml mouse cathepsin B (R&D Systems) 0.3 ml (group 3), or 0.5 *μ*g/ml cathepsin B 0.15 ml plus 5 *μ*g/ml mouse cystatin C (Sino Biological, Beijing, China) 0.15 ml (group 4) for a further 3 days. All mice were sacrificed on day 14.

After careful macroscopic inspection, the anterior walls were harvested and 5 mm tissue sections were fixed in 10% formalin and then embedded in paraffin. The sections were stained with hematoxylin and eosin and Masson's trichrome stains and examined in a blinded manner. The thickness of the submesothelial layer, defined as the area from the abdominal muscular surface to the peritoneal mesothelium, was measured at five random locations in each section. The mean submesothelial thickness was defined as the index for interstitial fibrosis. All procedures were carried under standard conditions in the Laboratory Animal Unit of Eulji University, and all experiments conformed to the Eulji University Institutional Animal Care and Use Committee (approval code: EUIACUC 11-13).

### 2.4. Statistical Analysis

The normality of the data distributions was tested by Kolmogorov–Smirnov tests. Skewed variables were log transformed or analyzed by nonparametric tests. Values with a normal distribution were expressed as mean ± standard deviation (SD), and non-normally distributed values were shown as medians and interquartile ranges (IQR). Correlations between two continuous variables were analyzed by Spearman's correlation to investigate the factors associated with PD effluent procathepsin B and cystatin C. All statistical analyses were performed using SPSS 17.0 (SPSS Inc., Chicago, IL, USA). *p* < 0.05 was considered statistically significant.

## 3. Results

### 3.1. Procathepsin B Levels Were Higher in Dialysis Effluent than in Serum in PD Patients

**Figure 1:**
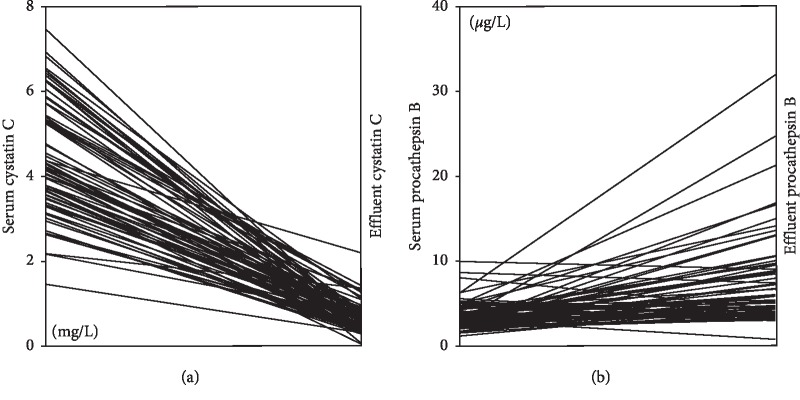
Individual variations in cystatin C (a) and procathepsin B (b) levels in serum and dialysate effluent. Cystatin C levels in serum were higher than in the dialysate effluent, while levels of procathepsin B showed the opposite pattern.

**Figure 2:**
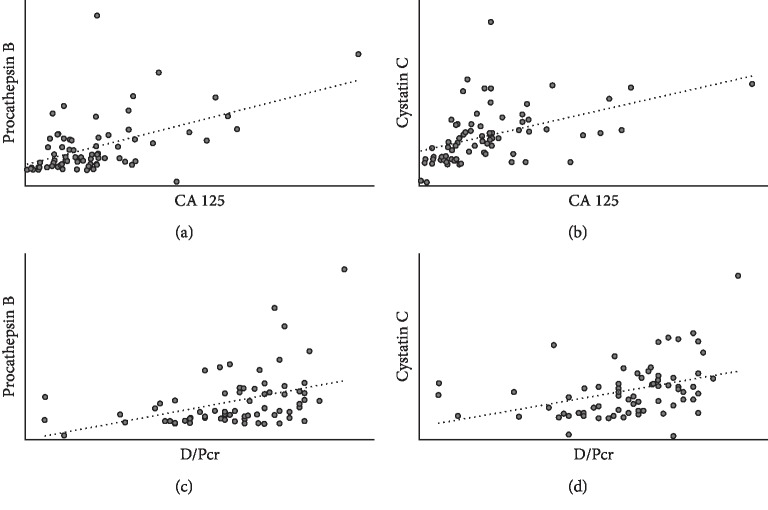
CA 125 correlation with procathepsin B (a) and cystatin C (b) and D/Pcr correlation with procathepsin B (c) and cystatin C (d) levels in PD effluent. Procathepsin B and cystatin C levels were positively correlated with CA 125 levels in PD effluent (*r*^2^ = 0.285, *p* < 0.001 and *r*^2^ = 0.444, *p* < 0.001, respectively). D/Pcr was also correlated with procathepsin B (*r*^2^ = 0.39, *p*=0.001) and cystatin C (*r*^2^ = 0.41, *p*=0.001) in PD effluent.

A total of 73 serum and dialysis effluent samples were obtained from 68 patients. The characteristics of the patients are shown in Table 1. The mean procathepsin B serum level was 3.6 *μ*g/L, and the median PD effluent level was 5.4 *μ*g/L. The mean cystatin C serum level was 4.4 mg/L, and the median PD effluent level was 0.6 mg/L. Cystatin C concentration was about 1000 times higher than procathepsin B concentration in serum and 100 times in the dialysate effluent. Cystatin C levels in serum were higher than in the dialysate effluent, while levels of procathepsin B showed the opposite pattern (Figure 1). Procathepsin B and cystatin C levels in PD effluent were not correlated with PD vintage (*r*^2^ = 0.009 and *r*^2^ = 0.01, respectively). But, procathepsin B and cystatin C levels were positively correlated with CA 125 levels in PD effluent (*r*^2^ = 0.285, *p* < 0.001 and *r*^2^ = 0.444, *p* < 0.001, respectively). D/Pcr was also correlated with procathepsin B (*r*^2^ = 0.39, *p*=0.0011) and cystatin C (*r*^2^ = 0.41, *p*=0.001) in PD effluent (Figure 2).

### 3.2. HPMCs Secreted Procathepsin B and Cathepsin B after High Glucose Stimulation

**Figure 3:**
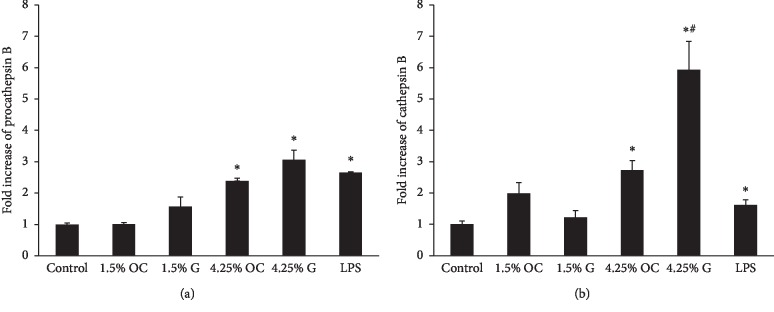
Procathepsin B (a) and cathepsin B (b) level in the supernatant of cultured human peritoneal mesothelial cells following various stimuli. Procathepsin B and cathepsin B level in the supernatant after the addition of various concentrations of mannitol and glucose and LPS are shown as fold increases compared with the control level in untreated cells. Procathepsin B and cathepsin B level was increased by 4.25% glucose and LPS. OC, *G*, and LPS means osmotic control, glucose, and lipopolysaccharide, respectively. ^*∗*^*p* < 0.05 compared with the control; ^#^*p* < 0.05 compared with its osmotic control.

Stimulation with 1.5% glucose and its osmotic control had no effect on procathepsin B secretion by HPMCs (1.6-fold; *p*=0.12 and 1.0-fold; *p*=0.86, respectively) (Figure 3(a)). However, treatment with 4.25% glucose and its osmotic control increased procathepsin B levels by 3.1-fold (*p*=0.01) and 2.4-fold (*p*=0.01), respectively. Procathepsin B level after stimulation with 4.25% glucose was higher than that of its osmotic control, but, statistically insignificant (*p*=0.09).

Cathepsin B in the supernatant of HPMCs was induced by 4.25% glucose and its osmotic control (5.9-fold; *p*=0.01 and 2.7-fold; *p*=0.01, respectively) but, not by 1.5% glucose stimulation (1.2-fold; *p*=0.41). 1.5% osmotic control increased the level of cathepsin B, but, statistically not significant (2.0-fold; *p*=0.05) (Figure 3(b)). 4.25% glucose increased the cathepsin B level 2 times higher than its osmotic control did (*p*=0.03). LPS also induced procathepsin B and cathepsin B secretion by HPMCs (2.7-fold; *p*=0.01 and 1.6-fold; *p*=0.04, respectively).

### 3.3. Cathepsin B Induced MMP-1, MMP-2, MMP-3, and TIMP-1 Secretion by HPMCs

**Figure 4:**
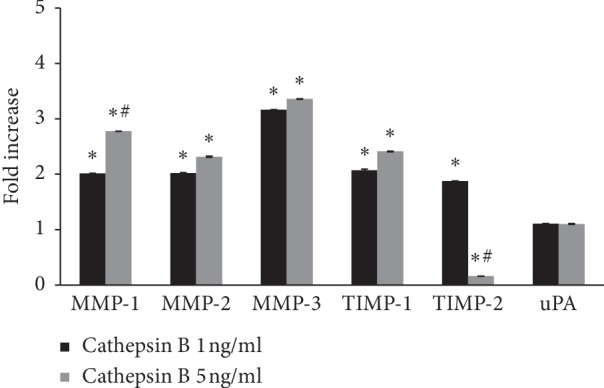
Protein expression of MMPs, TIMPs, and uPA in the supernatant of cultured human peritoneal mesothelial cells after treatment with cathepsin B. Cathepsin B increased MMP-1, MMP-2, MMP-3, and TIMP-1 protein expression, but had no effect on uPA. TIMP-2 was increased by 1 ng/ml cathepsin B but decreased by 5 ng/ml. MMP, TIMP, and uPA means matrix metalloproteinase, tissue inhibitor of metalloproteinase, and urokinase-type plasminogen activator, respectively. ^*∗*^*p* < 0.05 compared with protein expression without cathepsin B treatment, ^#^*p* < 0.05 compared with protein expression after1 ng/ml cathepsin B treatment.

Cathepsin B treatment induced the secretion of MMP-1, MMP-2, MMP-3, and TIMP-1 by HPMCs (Figure 4). Protein expression of MMP-1 was increased in dose-dependent manner. TIMP-2 secretion was increased by 1 ng/ml cathepsin B, but reduced by 5 ng/ml cathepsin B. uPA excretion by HPMCs was unaffected by cathepsin B treatment.

### 3.4. Cathepsin B Attenuated Peritoneal Fibrosis in a Murine Model

**Figure 5:**
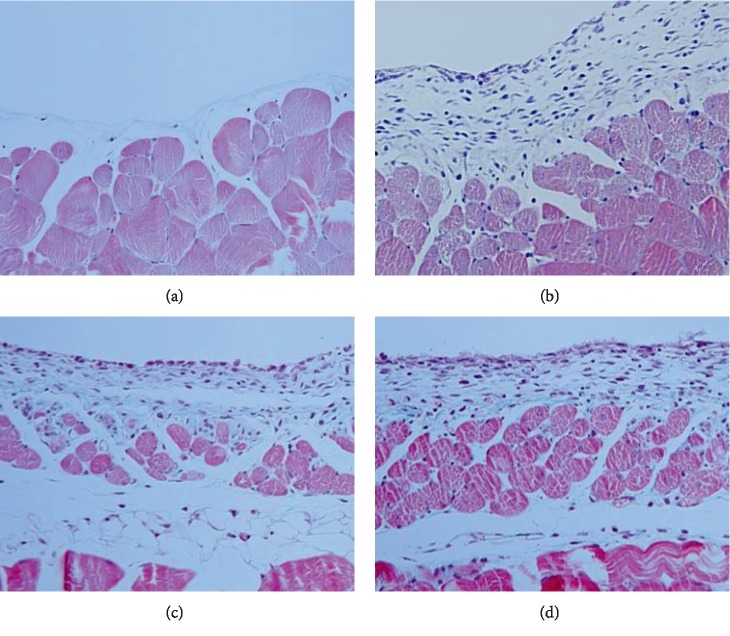
Microscopic examination of anterior abdominal wall in the mouse fibrosis model. Submesothelial thickness was increased from 31 ± 9 *μ*m in the control group (a) to 333 ± 59 *μ*m in the chlorhexidine-treated group (b). The addition of cathepsin B to the chlorhexidine-treated mice reduced the thickness of the submesothelial layer by 192 ± 25 *μ*m (c), while the simultaneous addition of cathepsin B and cystatin C had no significant effect (280 ± 37 *μ*m) (d).

Chlorhexidine treatment induced substantial peritoneal fibrosis after 14 days, as determined by microscopic examination (Figure 5). The submesothelial layer was 31 ± 9 *μ*m thick in the control group (group 1) compared with 333 ± 59 *μ*m in the chlorhexidine-treated group (group 2). Treatment of the fibrosis model mice with cathepsin B reduced the thickness of the submesothelial layer by 192 ± 25 *μ*m (group 3), while the simultaneous addition of cathepsin B and cystatin C had no significant effect (280 ± 37 *μ*m, group 4).

## 4. Discussion

This study provides the first evidence for cathepsin B secretion by HPMCs and for increased cathepsin B secretion following high glucose stimulation. Cathepsin B is a lysosomal cysteine protease, and its main function is the degradation of proteins that have entered the lysosomal system from outside the cell or from other compartments within the cell. The roles for cathepsin B in pathology may often be related to inappropriate location of the enzyme. Thus, the enzyme can be secreted as a proform procathepsin B and activated extracellularly [25].

The results of our clinical study showed that procathepsin B levels were lower than cystatin C levels in the serum and dialysate effluent from PD patients. However, procathepsin B levels in PD effluent were higher than in serum and were correlated with CA 125, which is a known marker of peritoneal cell mass [26]. These results suggest that procathepsin B was derived de novo from the peritoneal cavity, rather than being diffused from the blood. Cystatin C levels in the PD effluent were negligible, suggesting that it was not secreted by HPMCs. Accordingly, we did not measure cystatin C in the subsequent in vitro experiments.

Evidence for the effect of cathepsin B on peritoneal fibrosis is currently lacking. However, its role in ECM degradation suggests that it may have a protective function in peritoneal fibrosis. Cathepsin B was shown to promote the metastasis of cancer cells by degradation of the surrounding ECM, either directly or via activation of uPA, MMPs, and via inactivation of TIMPs [18–22]. In contrast, cathepsin B demonstrated profibrotic potential in rodent hepatic fibrosis models by inducing hepatocyte apoptosis and promoting hepatic stellate cell proliferation [27, 28]. Furthermore, inactivation of cathepsin B attenuated hepatic injury and fibrosis in mice [29–31]. In the current study, cathepsin B ameliorated the thickening of the submesothelial layer in a peritoneal fibrosis mouse model and its inhibitor, cystatin C, attenuated the effect of cathepsin B, suggesting a possible role for cathepsin B in reducing peritoneal fibrosis in patients receiving PD.

The mechanism whereby cathepsin B influences HPMCs is not clear. HPMCs can synthesize MMP-1, -2, -3, -8, and -9, along with TIMP-1 and -2. High glucose stimulation decreased MMP-1 and MMP-8 secretion and increased TIMP-1 and TIMP-2 in HPMCs [12, 13]. MMP-2 was associated with chemical peritoneal injury and showed increased levels in PD effluent in patients with peritoneal injury [14–16], while PD effluent levels of MMP-9 and TIMP-1 were higher in the onset of peritonitis [17]. We showed that cathepsin B treatment increased the secretion of both MMPs and TIMP-1, but not uPA, in HPMCs, indicating that the effect of cathepsin B on HPMCs might be mediated by MMP pathways. Administration of 4.25% glucose and LPS increased procathepsin B and cathepsin B secretion by HPMCs. Glucose remains the main osmotic material in physiologic peritoneal dialysate, and LPS, as a Gram-negative bacterial endotoxin, was used to mimic the environment of peritonitis. Previous studies showed that stimulation with high glucose and LPS upregulated the secretion of TGF-*β* and ECM by HPMCs [32–34]. The potentially beneficial effect of cathepsin B in the peritoneal fibrosis of our study suggests that its induction by high glucose and LPS might represent a negative feedback effect during the process of fibrosis. Our results also suggest that measurement of cathepsin B in PD effluent might be helpful in making an early diagnosis of peritonitis due to Gram-negative bacilli.

Our clinical study has limitations due to the small sample size and the cross-sectional nature which lacks longitudinal data. In the HPMCs experiment, we only measured the protein expression of MMPs, TIMPs, and uPA with cathepsin B stimulation. The effect of the addition of cystatin C should be evaluated. And, the expression of MMPs, TIMPs, and uPA should be confirmed in the mouse model. We also used the murine model that prevents generalization of the results to humans. Further studies are needed to study the role of cathepsin B in peritoneal fibrosis in PD patients.

## 5. Conclusions

The results of our in vivo study showing that cathepsin B decreased the thickness of the submesothelial layer suggest that cathepsin B, which is secreted by HPMCs after high glucose stimulation, might reduce peritoneal fibrosis in PD patients. Cathepsin B also increased the secretion of MMPs and TIMP-1, but not uPA, indicating that its effects in the peritoneum might be meditated by MMP pathways. Further studies are needed to confirm the effect of cathepsin B on the peritoneum and to explore the possibility of cathepsin B as a therapeutic target in PD patients with peritoneal fibrosis.

## Figures and Tables

**Table 1:** Characteristics of the patients.

Variables	Data^a^
Age (years)	59 ± 13
Sex, male/female (% of male)	35/33 (51%)
Diabetes mellitus, yes/no (%)	61/7 (90%)
PD vintage^b^ (days)	365 (124–983)
Urine volume^c^ (ml/day)	877 ± 662
Renal Kt/Vurea^c^	0.8 ± 0.8
Peritoneal Kt/Vurea	1.2 ± 0.5
Total Kt/Vurea	2.0 ± 0.8
D/Pcr	0.72 ± 0.12
Serum procathepsin B (*μ*g/L)	3.6 ± 1.6
Effluent procathepsin B (*μ*g/L)	5.4 (3.9–8.9)
Serum cystatin C (mg/L)	4.4 ± 1.3
Effluent cystatin C (mg/L)	0.6 (0.4–0.8)
Effluent CA 125 (IU/L)	41.3 (23.3–62.9)

^a^Data are expressed as mean ± SD, median (IQR), or %. ^b^Interval from PD catheter insertion to the day of obtaining serum and dialysis effluent samples. ^c^12 anuric cases are excluded.

## Data Availability

The data that support the findings of this study are available from the corresponding author upon reasonable request.
